# Diversity and convergence of mechanisms involved in pyrethroid resistance in the stored grain weevils, *Sitophilus* spp.

**DOI:** 10.1038/s41598-018-34513-5

**Published:** 2018-11-05

**Authors:** Khalid Haddi, Wilson R. Valbon, Luis O. Viteri Jumbo, Luiz O. de Oliveira, Raul N. C. Guedes, Eugenio E. Oliveira

**Affiliations:** 1 Departamento de Entomologia, Universidade Federal de Viçosa, Viçosa, MG 36570-900 Brazil; 2 Science without Border Program, Associate Researcher, Programa de Pós-Graduação em Entomologia, Universidade Federal de Viçosa, Viçosa, MG 36570-000 Brazil; 3 Departamento de Bioquímica e Biologia Molecular, Universidade Federal de Viçosa, Viçosa, MG 36570-900 Brazil; 4 USDA Agricultural Research Service, San Joaquin Valley Agricultural Sciences Center, Parlier, CA 93648 USA

## Abstract

Target-site mutations and changes in insect metabolism or behavior are common mechanisms in insecticide-resistant insects. The co-occurrence of such mechanisms in a pest strain is a prominent threat to their management, particularly when alternative compounds are scarce. Pyrethroid resistance among stored grain weevils (i.e., *Sitophilus* spp.) is an example of a long-standing concern, for which reports of resistance generally focus on a single mechanism in a single species. Here, we investigated pyrethroid resistance in maize and rice weevils (i.e., *Sitophilus zeamais* and *S*. *oryzae*), exploring potential knockdown resistance (*kdr*) mutations in their sodium channels (primary site for pyrethroid actions) and potential changes in their detoxification and walking processes. Resistance in pyrethroid-resistant rice weevils was associated with the combination of a *kdr* mutation (L1014F) and increases in walking and detoxification activities, while another *kdr* mutation (T929I) combined with increases in walking activity were the primary pyrethroid resistance mechanisms in maize weevils. Our results suggest that the selection of pyrethroid-resistant individuals in these weevil species may result from multiple and differential mechanisms because the L1014F mutation was only detected in Latin American rice weevils (e.g., Brazil, Argentina and Uruguay), not in Australian and Turkish rice weevils or Brazilian maize weevils.

## Introduction

The overuse of dichlorodiphenyltrichloroethane (i.e., DDT) up to the 1980’s and more recently of other synthetic insecticides (e.g., pyrethroids) for controlling stored product insect pests has contributed to the selection of insecticide-resistant strains, leading to severe economic losses in storage facilities worldwide. Regarding the pyrethroid insecticides, the resistance management is complicated because resistance occurs in a variety of forms, including reduced insecticide penetration, metabolic resistance (through detoxification enzymes), behavioral resistance and target-site alterations^1,2^. Although the pyrethroid insecticides exert their toxicity primarily by disrupting the function of the voltage-gated sodium channels in excitable cells^3–8^, these compounds also have secondary action targets (e.g., ionic imbalance and osmoregulatory dysfunction) that contribute to their activity^9–11^.

Multiple and distinct pyrethroid resistance mechanisms have been investigated in toxicological studies with focus on the contribution of the major mechanism, which includes target-site mutations (known as knockdown “*kdr*” resistance) and/or metabolic-based resistance^12–17^. The co-occurrence of distinct and multiple pyrethroid resistance mechanisms threatens resistance management strategies, with the threat particularly acute when alternative compounds are scarce, as is the case with stored grain weevils. Thus, it is essential to evaluate the potential of other classes of insecticides such as neonicotinoids, oxidiazines and spinosyns to control resistance populations of stored grain weevils.

Most of the losses in stored grains are caused by insect pests among which the grain weevils of the genus *Sitophilus* (e.g., the maize weevil *Sitophilus zeamais* Motsch. and the rice weevil *Sitophilus oryzae* L.) are particularly destructive^18,19^. The maize weevils, *Sitophilus zeamais* Motsch., and the rice weevil, *Sitophilus oryzae* L., are cosmopolitan and a major concern in tropical and subtropical regions, conditions that also occur in the Neotropical region^20,21^. Despite the economic importance of insecticide resistance in stored grain insect pests in general and grain weevils in particular^22,23^, studies on insecticide resistance are relatively limited for grain weevil species and do not usually explore the underlying molecular basis of the phenomenon^24–26^.

The mechanisms of pyrethroid resistance in the maize weevil *S*. *zeamais* as well as the fitness cost associated with it have been investigated^20,25,27,28^ but not those of the rice weevil. These studies with the maize weevil suggest that the primary resistance mechanism involves a single mutation in sodium channels (i.e., the *kdr* mutation T929I) that reduces the susceptibility to pyrethroids^27^, with secondary involvement of increased detoxification by glutathione-S-transferases^28^. However, this single mutation alone does not explain the high levels of resistance observed in maize weevil strains, and therefore, additional effort is required to understand the molecular basis of the resistance mechanisms involved in this species. The rice weevil is the subject of even greater neglect but also deserves attention because of the importance as a pest species and the relatively close phylogenetic relationship with the maize weevil^29–31^. Besides the resistance to insecticides resulting from the target site and metabolic alterations, other mechanisms associated with behavioral modification such as change in locomotory parameters have been reported in aphids^32^ and *Sitophilus* spp.^33,34^ but still need confirmation.

Thus, the present study was conducted to assess the physiological (e.g., occurrence of mutations in the sodium channel gene and activities of metabolic enzymes) and behavioral mechanisms (e.g., changes in walking patterns) of pyrethroid resistance in the maize and rice weevils (*S*. *zeamais* and *S*. *oryzae*, respectively). A series of toxicity, enzymatic, molecular and behavioral bioassays were conducted with a diverse and representative set of populations from both weevil species to achieve this objective. Our findings clearly demonstrated diversity and convergence of mechanisms involved in the pyrethroid resistance among strains of both species of grain weevils.

## Results

### Concentration-mortality bioassays

The probit model satisfactorily described the concentration-mortality data (goodness-of-fit tests exhibited low χ2-values [<9.5] and high *P*-values [>0.05]). The resistance ratios were estimated relative to the LD_50_ for the most susceptible strain for each insecticide (Tables 1 and 2). Based on the LD_50_ values obtained for the 14 maize weevil strains, the pyrethroid lambda-cyhalothrin and the neonicotinoid thiamethoxam were the most potent (i.e. lowest LD_50_ values) insecticides followed by the neonicotinoid imidacloprid and the spynosin spinosad (Table 1). Furthermore, the most susceptible maize weevil strain varied with insecticide. Individuals from E. S. Pinhal-SP were the most susceptible to both neonicotinoid insecticides (i.e., thiamethoxam and imidacloprid); while individuals from Teresina-PI (for the pyrethroid lambda-cyhalothrin) and Cristalina-GO (for the spynosin spinosad) were the most susceptible to other insecticides (Table 1). Regarding the pyrethroid insecticide lambda-cyhalothrin, and based on the 95% confidence intervals for resistance ratios (RR), five strains (total of 14) exhibited moderate to high resistance (i.e., RR > 5.0; Table 1). No resistance was found for spinosad, with the resistance ratios (RR) all below 2.8. Regarding the neonicotinoid insecticides, only three populations (Amambai-MS, Piracicaba-SP and Sao João-PR) exhibited (low) resistance levels to imidacloprid with resistance ratios (RR) between 2.7 and 3.6, while six populations (Amambai-MS, Balsas-MA, Ipojuca-PE, Jacarezinho-PR, Juiz de Fora-MG and Piracicaba-SP) exhibited low levels of thiamethoxam resistance (RR between 2.5 and 3.8). Generally, resistance to both neonicotinoids was either absent or very low among the strains tested.

**Table 1:** Relative toxicity of four insecticides (i.e., lambda-cyhalothrin, imidacloprid, thiamethoxam and spinosad) to 14 Brazilian strains of *S*. *zeamais*.

Insecticide type	*S*. *zeamais* strains	No. of insects	LD_50_ (95% FI^a^) mg a.i/kg of grains	LD_95_ (95% FI) mg a.i/kg of grains	RR_50_ (95% CL)^b^	*Χ* ^2^	*P*
Lambda-cyhalothrin	Amambai-MS	519	1.6 (1.3–1.8)	7.8 (6.2–10.5)	5.4 (4.0–7.3)	6.05	0.11
	Balsas-MA	521	0.7 (0.6–0.9)	1.6 (1.3–2.3)	2.6 (1.9–3.4)	6.31	0.10
	Barreiras-BA	629	1.0 (0.9–1.1)	4.7 (3.8–6.3)	3.5 (2.6–4.8)	4.23	0.38
	Canarana–MT	420	0.8 (0.7–0.9)	2.6 (2.2–3.2)	2.8 (2.1–3.8)	1.03	0.60
	Cristalina-GO	318	0.7 (0.2–1.2)	3.2 (2.5–4.9)	2.6 (1.7–4.0)	2.28	0.56
	E. S. Pinhal-SP	521	0.9 (0.7–1.1)	2.5 (1.7–6.2)	3.1 (2.3–4.3)	8.62	0.10
	Ipojuca-PE	417	2.0 (1.7–2.3)	8.7 (6.9–11.7)	7.1 (5.3–9.7)	1.77	0.41
	Iragassu-PE	629	1.0 (0.9–1.1)	2.4 (2.1–2.8)	3.6 (2.7–4.9)	4.51	0.34
	Jacarezinho-PR (SzPyrR1)	627	2.8 (2.4–3.2)	17.6 (13.6–24.4)	9.9 (7.3–13.5)	5.59	0.24
	Juiz de Fora-MG(SzPyrR2)	423	5.4 (4.9–6.2)	16.7 (13.7–21.8)	19.5 (14.4–26.4)	3.96	0.14
	Piracicaba-SP	515	0.9 (0.8–1.0)	2.0 (1.7–2.4)	3.4 (2.5–4.5)	3.43	0.33
	Sao João-PR	630	1.2 (1.0–1.4)	12.9 (8.7–22.5)	4.3 (3.1–5.8)	2.85	0.58
	Teresina-PI	415	0.3 (0.2–0.4)	4.1 (2.7–7.7)	*	2.64	0.24
	Xapuri-AC	526	1.0 (0.9–1.1)	2.0 (1.8–2.5)	3.6 (2.7–4.9)	3.68	0.30
	Sete Lagoas–MG (SzSusc)	526	0.71 (0.7–0.8)	1.5 (1.3–1.7)	2.6 (1.9–3.5)	4.31	0.23
Imidacloprid	Amambai-MS	622	4.3 (3.5–5.2)	53.1 (34.2–104.5)	2.7 (1.8–4.1)	2.04	0.73
	Balsas-MA	405	2.3 (1.6–2.9)	22.7 (15.8–39.6)	1.4 (0.9–2.2)	3.64	0.70
	Barreiras-BA	507	2.2 (1.5–2.7)	38.4 (22.9–92.7)	1.3 (0.9–2.0)	6.17	0.10
	Canarana–MT	399	1.9 (0.9–3.6)	12.5 (5.6–21.9)	1.2 (0.6–2.3)	2.73	0.26
	Cristalina-GO	420	1.9 (1.7–2.5)	13.4 (11.7–15.2)	1.2 (0.9–2.5)	4.85	0.31
	E. S. Pinhal-SP	619	1.6 (0.8–2.4)	11.3 (4.9–28.7)	*	3.00	0.56
	Ipojuca-PE	409	3.1 (2.3–4.0)	48.3 (29.2–108.4)	1.9 (1.3–3.0)	2.54	0.76
	Iragassu-PE	619	2.4 (1.7–2.9)	34.1 (22.6–65.0)	1.5 (1.0–2.2)	2.93	0.57
	Jacarezinho-PR (SzPyrR1)	510	1.7 (1.2–2.2)	11.9 (8.0–24.9)	1.1 (0.6–2.0)	2.42	0.49
	Juiz de Fora-MG (SzPyrR2)	414	2.7(1.9–3.5)	61.1 (34.1–161.4)	1.7 (1.1–2.6)	2.02	0.36
	Piracicaba-SP	617	5.7 (4.8–6.8)	76.5 (46.8–162.0)	3.6 (2.3–5.6)	1.97	0.91
	Sao João-PR	623	5.1 (4.4–5.9)	47.3 (32.7–80.6)	3.2 (2.1–4.8)	4.03	0.40
	Teresina-PI	607	2.0 (1.4–2.6)	39.6 (25.08–82.8)	1.3 (0.8–1.9)	2.29	0.68
	Xapuri-AC	509	2.6 (2.0–3.1)	33.4 (21.2–70.2)	1.6 (1.1–2.4)	2.91	0.41
	Sete Lagoas–MG (SzSusc)	617	1.21 (0.9–1.7)	13.0 (6.6–41.9)	0.8 (0.5–1.2)	3.82	0.43
	SzPyrSel	496	1.83 (0.7–3.4)	15.5 (9.6–18.3)	0.5 (0.34–0.78)	5.63	0.13
Thiamethoxam	Amambai-MS	736	0.85 (0.7–1.1)	8.4 (4.8–18.9)	2.5 (2.1–3.0)	8.71	0.12
	Balsas-MA	523	1.07 (0.97–1.3)	7.2 (4.6–14.3)	3.1 (2.7–3.6)	5.52	0.14
	Barreiras-BA	493	0.46 (0.4–0.5)	2.7 (2.1–3.8)	1.4 (1.2–1.6)	4.64	0.20
	Canarana–MT	502	0.70 (0.6–0.8)	1.5 (1.3–2.1)	2.1 (1.8–2.3)	1.87	0.60
	Cristalina-GO	501	0.59 (0.5–0.7)	5.9 (4.1–9.9)	1.7 (1.5–2.1)	4.38	0.22
	E. S. Pinhal-SP	598	0.34 (0.3–0.4)	2.2 (1.8–2.9)	*	4.67	0.32
	Ipojuca-PE	605	1.29 (1.1–1.5)	7.0 (4.9–12.7)	3.8 (3.3–4.3)	2.86	0.41
	Iragassu-PE	679	0.50 (0.4–0.6)	2.9 (2.0–5.1)	1.5 (1.3–1.7)	9.50	0.09
	Jacarezinho-PR (SzPyrR1)	595	0.87 (0.8–0.9)	4.9 (3.7–7.5)	2.6 (2.3–2.9)	4.28	0.37
	Juiz de Fora-MG (SzPyrR2)	499	1.25 (1.1–1.5)	8.1 (5.5–14.4)	3.7 (3.2–4.2)	2.26	0.52
	Piracicaba-SP	622	0.98 (0.8–1.1)	9.6 (6.3–17.9)	2.9 (2.5–3.3)	3.44	0.49
	Sao João-PR	595	0.39 (0.3–0.5)	2.3 (1.9–3.0)	1.2 (1.0–1.3)	4.71	0.32
	Teresina-PI	595	0.49(0.4–0.6)	2.8 (2.3–3.8)	1.5 (1.3–1.7)	1.92	0.75
	Xapuri-AC	494	0.56 (0.4–0.74)	1.8 (1.30–4.42)	1.6 (1.4–1.9)	7.16	0.07
	Sete Lagoas–MG (SzSusc)	497	0.68 (0.6–0.8)	2.6 (2.1–3.7)	2.1 (1.8–2.3)	2.79	0.43
	SzPyrSel	404	1.46 (1.1–2.0)	15.6 (8.4–41.9)	4.3 (3.2–5.7)	2.21	0.33
Spinosad	Amambai-MS	612	12.3(10.6–13.7)	49.2 (39.3–70.1)	1.3 (1.2–1.4)	7.68	0.10
	Balsas-MA	504	16.4(18.5–18.0)	63.8 (48.0–102.3)	1.7 (1.5–1.9)	5.71	0.13
	Barreiras-BA	520	13.1(8.8–16.1)	228.1 (99.2–372)	1.4 (0.8–2.5)	1.85	0.61
	Canarana–MT	627	11.9(10.5–13.0)	36.1 (31.1–45.0)	1.2 (1.1–1.3)	6.28	0.18
	Cristalina-GO	595	9.60 (8.3–10.8)	32.6(26.9–43.2)	*	1.89	0.76
	E. S. Pinhal-SP	418	27.3 (22.5–32.6)	107.1 (58.7–195.2)	1.1 (1.0–1.3)	7.94	0.10
	Ipojuca-PE	519	24.7 (21.1–30.4)	62.6(41.0–109.1)	0.9 (0.8–1.0)	6.85	0.08
Spinosad	Igarassu-PE	525	22.7(21.3–24.6)	53.0 (45.0–66.6)	2.4 (2.2–2.6)	4.14	0.25
	Jacarezinho-PR (SzPyrR1)	632	30.7 (25.5–36.9)	293.7 (134.9–354.1)	0.9 (0.7–1.0)	1.23	0.87
	Juiz de Fora-MG (SzPyrR2)	614	27.2 (23.4–32.4)	154.4 (91.7–416.7)	2.8 (2.1–3.9)	3.99	0.14
	Piracicaba-SP	615	18.8 (15.2–20.8)	83.0 (60.2–142.1)	2.0 (1.7–2.3)	4.29	0.37
	Sao João-PR	519	19.5(17.4–22.2)	98.6 (65.5–207.1)	2.0 (1.7–2.5)	2.01	0.57
	Teresina-PI	526	19.3(15.9–22.7)	45.7 (33.4–124.8)	2.0 (1.8–2.2)	6.90	0.08
	Xapuri-AC	496	20.4 (19.2–21.7)	42.6 (37.5–50.42)	2.1(2.0–2.3)	3.76	0.29
	Sete Lagoas–MG (SzSusc)	635	15.7 (14.5–16.9)	37.3 (32.5–45.5)	1.6 (1.5–1.8)	7.36	0.12
	SzPyrSel	522	23.6 (21.2–27.7)	37.3 (32.5–45.5)	2.5 (2.0–3.0)	6.07	0.11

^a^FI = Fiducial Intervals; ^b^RR_50_ = Resistance ratio determined by LD_50_ of given population/LD_50_ of most susceptible strain (note the asterisks for each insecticide type); 95% CL = 95% Confidence limits; *χ*^2^ = *Chi*-square for lack-of-fit to the probit model, and *P* = Probability associated with the *chi*-square statistic. SzPyrSel = the *S*. *zeamais* laboratory strain selected for pyrethroid resistance.

**Table 2:** Relative toxicity of five insecticides to two strains of *S*. *zeamais* [i.e., the pyrethroids susceptible (SzSusc) and pyrethroid resistant (SzPyrSel) strains] and two strains of *S*. *oryzae* (i.e., SoPyrTol and SoPyrR).

Insecticide	*Sitophilus* strains	No. of insects	LD_50_ (95% FI) mg a.i/kg of grains	LD_95_ (95% FI) mg a.i/kg of grains	RR_50_ (95% CL)	RR^a^_50_ (95% CL)	*Χ* ^2^	*P*
Deltamethrin	SzSusc	493	0.68 (0.5–0.9)	1.6 (1.2–2.8)	*	*	7.42	0.09
	SzPyrSel	(—)	(—)	(—)	(>2500)	(>2500)	(—)	(—)
	SoPyrTol	490	25.95 (23.1–28.5)	82.5 (69.3–106.7)	*	37.6 (35.1–40.3)	2.20	0.53
	SoPyrR	495	69.39 (64.2–76.0)	201.8 (159.8–291.5)	2.7 (2.4–2.9)	101.0 (91.2–110.9)	1.54	0.67
Lambda-Cyhalothrin	SzSusc	526	0.71 (0.7–0.8)	1.5 (1.3–1.7)	*	*	4.31	0.23
	SzPyrSel	(—)	(—)	(—)	(—)	(—)	(—)	(—)
	SoPyrTol	596	12.06 (10.1–13.9)	54.4(45.3–69.1)	*	16.9 (15.9–18.0)	1.57	0.81
	SoPyrR	588	174.3 (146.1–219.6)	1243.1(973.4–2259.0)	14.5 (9.8–21.2)	244.6 (167.5–357.3)	4.46	0.61
Indoxacarb	SzSusc	689	0.40 (0.3–0.5)	5.2 (3.9–7.4)	*	*	6.77	0.24
	SzPyrSel	599	3.79 (3.1–4.5)	15.3 (12.9–19.1)	9.5 (7.8–11.5)	9.5 (7.8–11.5)	1.67	0.80
	SoPyrTol	496	3.05 (2.6–3.5)	11.2 (9.3–14.2)	*	7.6 (6.3–9.6)	1.06	0.78
	SoPyrR	512	1.55 (1.2–1.9)	14.2 (10.6–20.5)	0.5 (0.5–0.6)	3.9 (3.2–4.8)	4.72	0.45
Imidacloprid	SzSusc	617	1.21 (0.9–1.7)	13.0 (6.6–41.9)	*	*	3.82	0.43
	SzPyrSel	496	0.83 (0.7–3.4)	15.5 (9.60–18.3)	0.7 (0.5–1.1)	0.7 (0.5–1.1)	5.63	0.13
	SoPyrTol	398	1.32 (0.2–5.2)	14.2 (8.50–19.4)	*	1.1 (0.17–2.3)	1.40	0.49
	SoPyrR	450	1.54 (0.5–4.1)	16.6 (11.2–25.1)	1.2 (0.6–5.1)	1.3 (0.27–2.1)	1.34	0.85
Thiamethoxam	SzSusc	497	0.68 (0.6–0.8)	2.6 (2.1–3.7)	*	*	2.79	0.43
	SzPyrSel	404	1.46 (1.1–2.0)	15.6 (8.4–41.9)	2.2 (1.7–2.8)	2.2 (1.7–2.8)	2.21	0.33
	SoPyrTol	597	1.95 (1.5–2.9)	25.3 (11.9–90.9)	*	2.9 (1.9–4.2)	4.10	0.39
	SoPyrR	449	0.94 (0.69–1.2)	12.8 (7.3–38.2)	0.48 (0.3–0.7)	1.4 (1.1–1.7)	3.12	0.53
Spinosad	SzSusc	635	15.7 (14.5–16.9)	37.3 (32.5–45.5)	*	*	7.36	0.12
	SzPyrSel	522	23.6 (21.2–27.3)	80.2 (57.9–141.6)	1.5 (1.2–1.8)	1.5 (1.2–1.8)	6.07	0.11
	SoPyrTol	499	17.2 (13.8–20.7)	80.5 (64.6–107.5)	*	1.1 (0.7–1.3)	1.88	0.59
	SoPyrR	512	21.5 (19.3–26.6)	76.1 (69.7–82.4)	1.25 (0.9–8.3)	1.3 (1.2–1.7)	4.28	0.34

FI = Fiducial Intervals; RR_50_ = Resistance ratio determined by LD_50_ of given population/LD_50_ of most susceptible population (*); RR^a^_50_ = Resistance ratio determined by LD_50_ of given population/LD_50_ of SzSusc; 95% CL = 95% Confidence limits; *χ*^2^ = *Chi*-square for lack-of-fit to the probit model, and *P* = Probability associated with the *chi*-square statistic. (—) Data not available as no dose-response curve could be computed for the selected population SzPyrSel.

The comparisons of the pyrethroid susceptibilities between the rice weevil strains (i.e., SoPyrTol and SoPyrR) revealed differential susceptibilities to deltamethrin (RR = 2.7 [2.2–3.3]-fold) and lambda-cyhalothrin (RR = 14.5 [10.2–20.6]-fold). When these levels of pyrethroid susceptibility in rice weevils were compared with that of the SzSusc strain (i.e., the *S*. *zeamais* strain that is pyrethroid susceptibility pattern), their differential susceptibility to pyrethroids was more evident (Table 2). Furthermore, the pyrethroid-selected strain of maize weevils (SzPyrSel: RR = 9.5 [7.8–11.5]) and of rice weevils (SoPyrTol: RR = 7.6 [6.3–9.6] and SoPyrR: RR = 3.9 [3.2–4.8]) exhibited moderately high resistance ratios to indoxacarb. Both neonicotinoid (i.e., thiamethoxam and imidacloprid) and spinosad insecticides exhibited high toxicity to the four strains, regardless of their pyrethroid resistance status.

### knock-down (*kdr*) mutations conferring pyrethroids resistance

The use of degenerate primers designed against conserved regions of *para* sodium channel gene sequences of different insects species, followed by the use of specific primers designed based on the obtained sequences, allowed the amplification and cloning of total fragments of 6129 and 6024 bp of the *para* sodium channel genes of the maize and rice weevils, respectively (GenBank accession: *S*. *zeamais:* MG813771; *S*. *oryzae*: MG813770). The encoded amino acid sequences of these fragments are available in Supplementary Fig. 1. The analysis of sequence homology using the BLAST interface (NCBI) showed the total sequenced fragments had high similarity (over 90% direct amino acid identity) to other insects particularly among coleopterans, including the red flour beetle *Tribolium castaneum*, the mountain pine beetle *Dendroctonus ponderosae* and the pollen beetle *Meligethes aeneus*.

**Figure 1:**
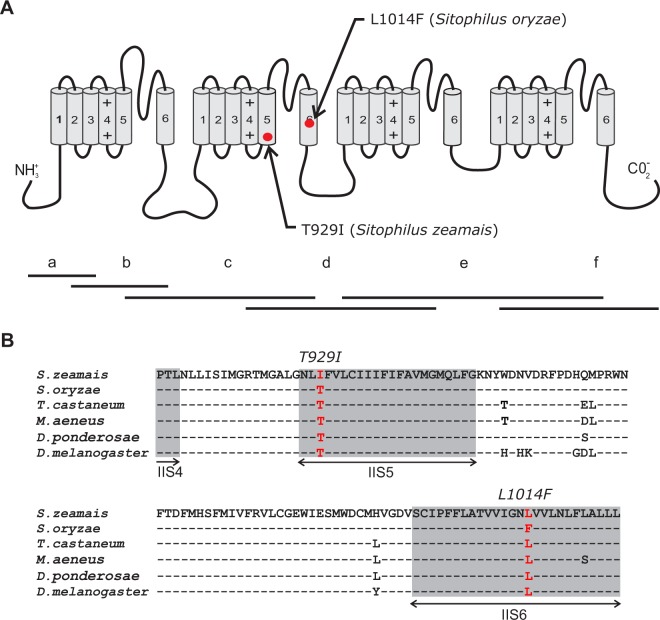
Pyrethroid resistance-associated amino acid substitutions in sodium channels of *Sitophilus spp* weevils. (**A**) Schematic representation of insect voltage-gated sodium channels (VGSC) indicating two knockdown (*kdr*) resistance mutations (T929I and L1014F) as well as the positions of the sequenced fragments (a-f). The sodium channel protein is consisted of four homologous repeats (I - IV), each having six transmembrane segments (S1-S6). The isoleucine, phenylalanine and methionine motif (IFM) has a crucial role in the VGSC inactivation. Residue positions correspond to the house fly numbering (GenBank number: X96668). (**B**) Alignment of the deduced amino acid sequences of the region encompassing Domain II S5-S6 (in grey) of *S*. *zeamais* and *S*. *oryzae* with *Drosophila melanogaster para* sodium channel proteins. Dashes represent identical residues. The positions of the encountered mutations (T929 and F1014) are in Red.

The sequenced fragments from both grain weevil species contained features that are characteristic of voltage gated sodium channels, including four homologous domains with each domain containing six transmembrane segments: (1) the voltage sensor region S4 of each domain has four to seven basic amino acid residues, arginine or lysine, separated by two neutral amino acid residues^35^; (2) four conserved amino acid residues (DEKA) located in loops between S5 and S6 of domains I, II, III and IV, respectively, and known to be critical for sodium selectivity^36^; and (3) the conserved motif (MFM) found at the linker between domains III and IV of sodium channels and playing a critical role in the fast inactivation of the channel^37^. The examination of the cDNA sequences from pooled individuals revealed the existence of only two mutations within the domain II region that were previously associated with *kdr* resistance in several other insect species (Fig. 1). One point mutation resulting from a leucine to phenylalanine (L1014F) amino acid substitution was found at the IIS6 region for *S*. *oryzae*, and one point mutation within the same region that resulted in a threonine to isoleucine mutation (T929I) was found within IIS5 for *S*. *zeamais* (numbering is based on the housefly *Musca domestica* sequence).

Specific primers (SZ2 and SZ3) were used in the analysis of genomic DNA of fifteen individual insects from each population to screen for the existence/absence of the mutations found in the populations used (Table 3). The results showed that the T929I mutation was present and homozygous in all fifteen individuals sequenced from the SzPyrSel strain, whereas the mutation was present as heterozygous in lower frequencies only in the two other Brazilian strains of *S*. *zeamais* (i.e., SzPyrR1 and SzPyrR1). The mutation T929I was not found in the other strains of *S*. *zeamais* screened in our study. The L1014F mutation was present in all strains of *S*. *oryzae* from Brazil, Argentina and Uruguay but was not detected in the strains of either Australia or Turkey (Table 3). This mutation was homozygous in only two strains (Viçosa1-MG and São Borja-RS).

**Table 3:** Mutation frequencies in stored grain weevils (i.e., *Sitophilus* spp.) collected from different locations around the world.

Country	Strain	Species	Mutation frequency	
			L1014F	T929I
Brazil	Sete Lagoas-MG (SzSusc)	*S*. *zeamais*	0.0	0.0
Brazil	SzPyrSel	*S*. *zeamais*	0.0	1.0
Brazil	Jacarezinho-PR (SzPyrR1)	*S*. *zeamais*	0.0	0.25
Brazil	Juiz de Fora-MG (SzPyrR2)	*S*. *zeamais*	0.0	0.15
Brazil	Cascavel-PR (SoPyrTol)	*S*. *oryzae*	0.65	0.0
Brazil	Viçosa-MG	*S*. *oryzae*	0.50	0.0
Brazil	Viçosa1-MG	*S*. *oryzae*	1.00	0.0
Brazil	Volta Redonda-RJ	*S*. *oryzae*	0.25	0.0
Brazil	São Borja-RS	*S*. *oryzae*	1.0	0.0
Brazil	Porto Nacional-TO	*S*. *oryzae*	0.5	0.0
Argentina	SoPyrR	*S*. *oryzae*	0.65	0.0
Uruguay	Uruguay	*S*. *oryzae*	0.5	0.0
Australia	QQSO1537 (Moura Queensland)	*S*. *oryzae*	0.0	0.0
Australia	QQSO1543 (Springsure Queensland)	*S*. *oryzae*	0.0	0.0
Australia	QTSO1 (Tasmania)	*S*. *oryzae*	0.0	0.0
Australia	QQSO16 (South Australia)	*S*. *oryzae*	0.0	0.0
Turkey	Trukey1	*S*. *oryzae*	0.0	0.0
Turkey	Turkey2	*S*. *oryzae*	0.0	0.0

### Metabolic resistance to pyrethroid in rice but not in maize weevils

**Figure 2:**
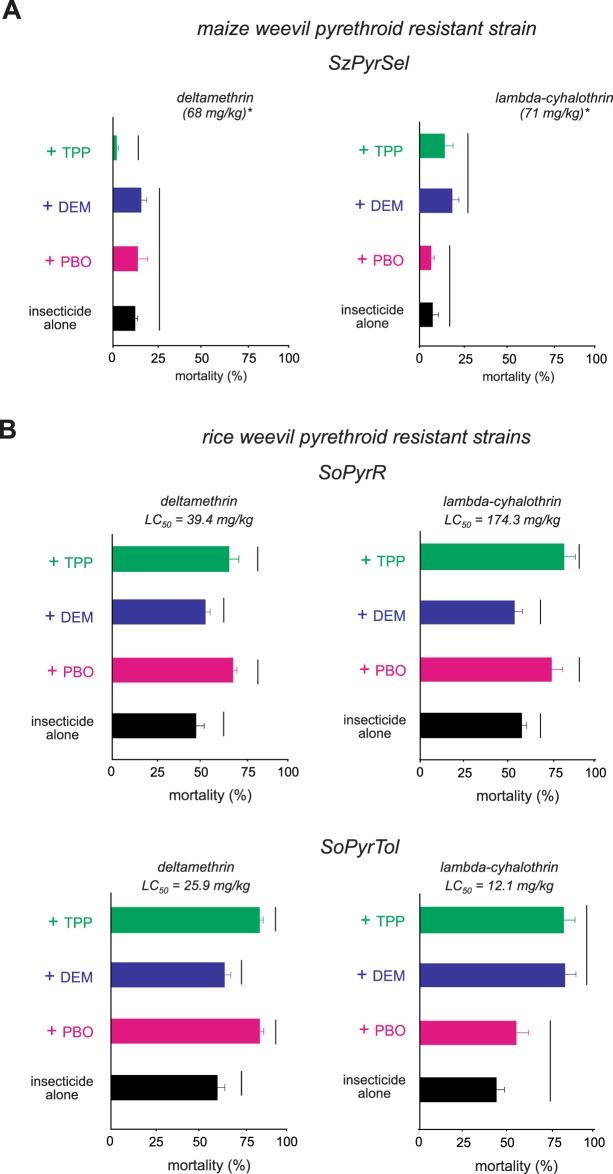
Effects of synergized pyrethroids in the mortality of adult *Sitophilus spp* weevils. (**A**) Comparative effects of the synergists piperonyl butoxide (PBO), diethyl maleate (DEM), and triphenyl phosphate (TPP) on the mortality caused by deltamethrin and lambda-cyhalothrin on the weevil strains that were resistant (i.e., the *S*. *zeamais* SzPyrSel strain and the *S*. *oryzae* SoPyrTol and SoPyrR strains) to pyrethroid insecticides. Asterisks indicated that this pyrethroid concentration is 100-fold higher than the estimated for the *S*. *zeamais* SzSusc strain (i.e., the pyrethroid susceptibility pattern strain). (**A**,**B**) Treatments lined with the same vertical bar do not differ statistically (paired *t*-test, *P* < 0.05).

The bioassays with synergists (i.e., PBO, TPP, and DEM) revealed distinct involvement of the detoxification enzymes among the weevil strains (i.e., SzPyrSel, SoPyrR and SoPyrTol) that expressed a *kdr* mutation (Fig. 2). The mortality recorded for the synergists alone (at 1 mg/mL) was never higher than 3%, not differing from the negative control (i.e., insects not exposed to any insecticide or synergists). For the maize weevil strain highly resistant to pyrethroids SzPyrSel (i.e., expressing the *kdr* mutation T929I), the mortality levels obtained for the synergized insecticides were never higher than 25%, even at pyrethroid concentrations 100-fold higher than the LD_50_ for the maize weevil susceptible strain (SzSusc) (Fig. 2A). However, it is worth to note that the synergists DEM and TPP significantly increased (*F* = 5.78; *df* = 6; *P* = 0.001) the mortality caused by lambda-cyhalothrin while TPP significantly reduced (*F* = 4.63; *df* = 6; *P* = 0.0023) the mortality by deltamethrin in SzPyrSel (Fig. 2A).

Regarding the pyrethroid-resistant rice weevil strains, while PBO and TPP synergists significantly increased the mortality caused by deltamethrin (*F* = 25.14; *df* = 6; *P* = 0.0002) and lambda-cyhalothrin (*F* = 16.9; *df* = 6; *P* = 0.003) in the SoPyrR strain (Fig. 2B), the mortality levels of SoPyrTol increased when both pyrethroids (i.e., deltamethrin [*F* = 125.21; *df* = 6; *P* = 0.0001] and lambda-cyhalothrin [*F* = 44.94; *df = *6; *P* = 0.0001]) were synergized with TPP (Fig. 2C). Furthermore, high mortality was also recorded for SoPyrTol individuals when deltamethrin was synergized by PBO and when lambda-cyhalothrin was synergized by DEM (Fig. 2C). However, even with synergist use, the complete suppression of pyrethroid resistance was not achieved (i.e., 100% mortality was not reached).

### Enhanced detoxification in pyrethroid resistant rice weevils but not in resistant maize weevils

**Figure 3:**
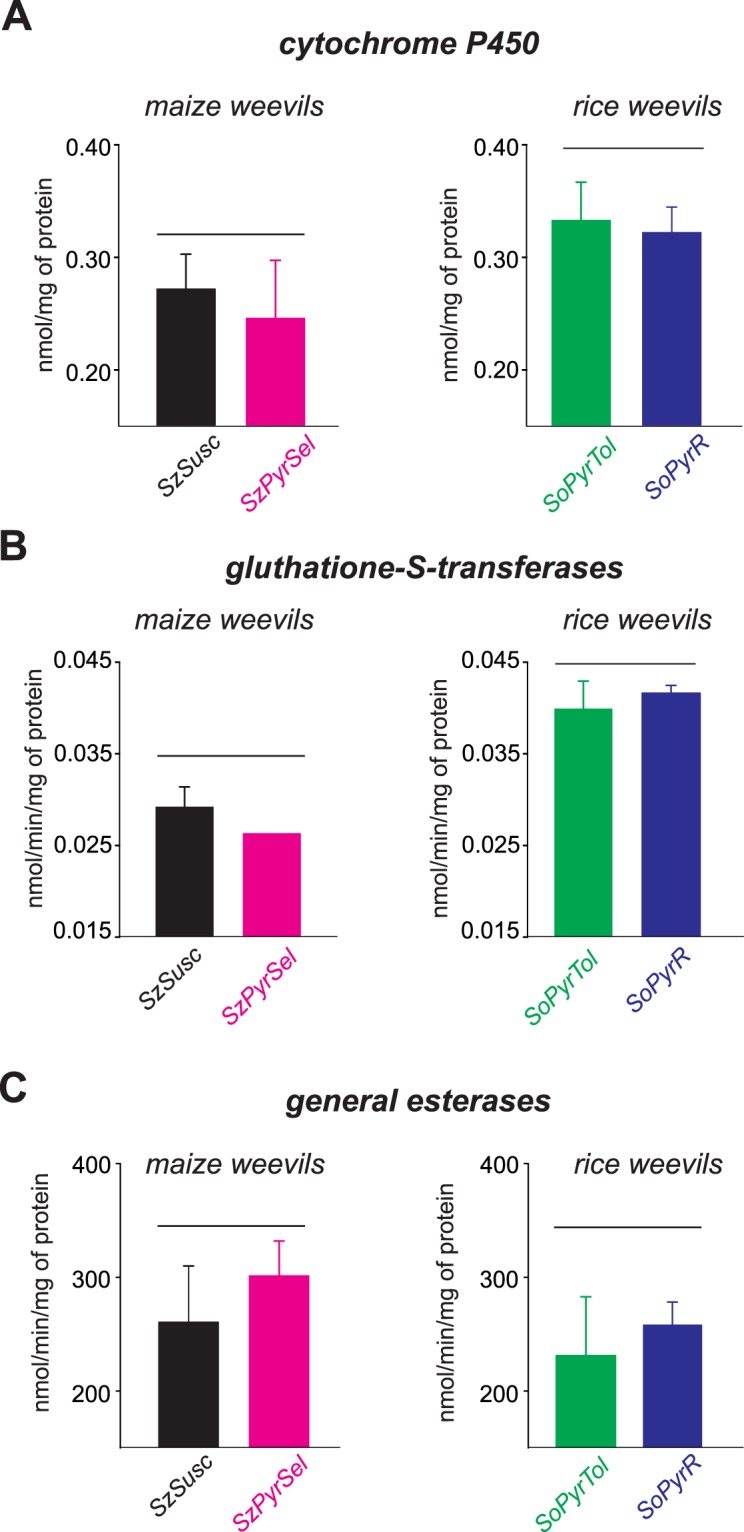
Activity profiles of detoxification enzymes in pyrethroid resistant *Sitophilus spp* weevils. Activities of the enzymes cytochrome P450 (**A**), glutathione-S-transferases (**B**) and general esterases (**C**) measured in adults insects of the *S*. *zeamais* SzSusc strain (i.e., the pyrethroid susceptibility pattern strain) and of the weevils strains that were resistant (i.e., the *S*. *zeamais* SzPyrSel strain and the *S*. *oryzae* SoPyrTol and SoPyrR strains) to pyrethroid insecticides. Each bar corresponds to the mean of five independent assays (±SE). Histograms under the same horizontal bar do not differ statistically (Tukey test, *P* < 0.05).

The oxidase (*df* = 3; *F* = 9.8; *P* = 0.005) and GST (*df* = 3; *F* = 0.7; *P* = 0.04) activities only exhibited significant differences between weevil species (i.e., *S*. *zeamais* and *S*. *oryzae*) (Fig. 3A,B). However, when the activities of these enzymes were compared between strains of the same species (i.e., between SzSusc and SzPyrSel for maize weevils and between SoPyrTol and SoPyrR for rice weevils), no significant differences were observed (Fig. 3A,B). Furthermore, no significant differences were observed for the activity of general esterases (*df* = 3; *F* = 1.8; *P* = 0.22; Fig. 3C).

### Altered and divergent walking activity in pyrethroid resistant weevil species

**Figure 4:**
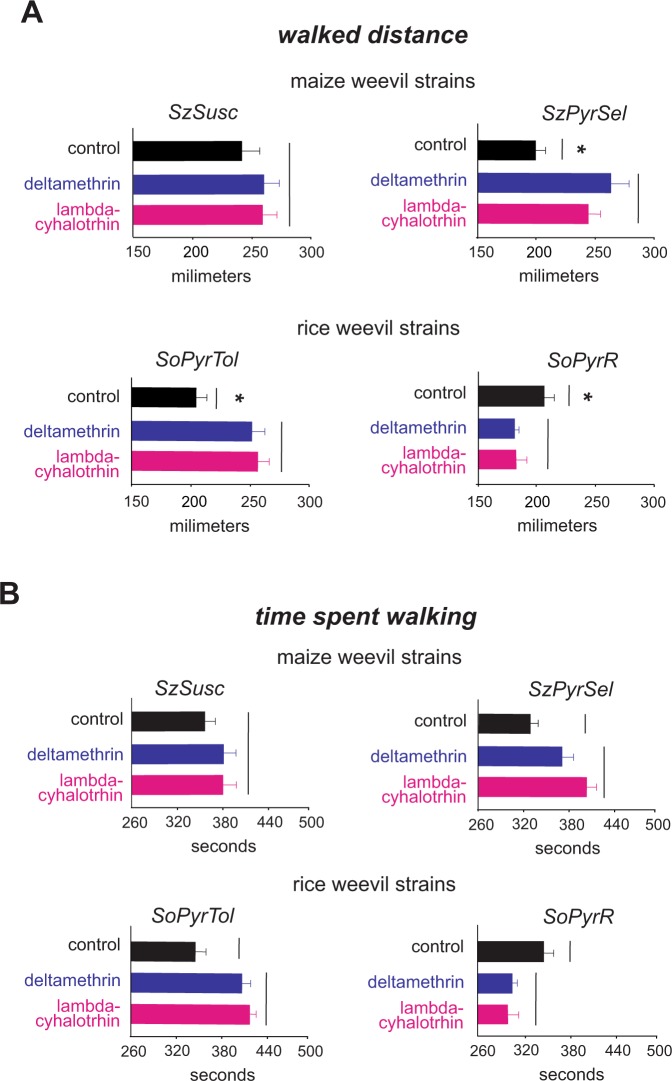
Pyrethroid induced changes in locomotion of *Sitophilus spp* weevils. Walked distance (**A**) and time spent walking (**B**) by pyrethroid susceptible (SzSusc) and pyrethroid resistant weevil strains (i.e., the *S*. *zeamais* SzPyrSel strain and the *S*. *oryzae* SoPyrTol and SoPyrR strains) in arenas fully treated and untreated with deltamethrin or lambda-cyhalothrin. Each histogram bar represents the mean (±SE) of 20 replicates. Bars lined with the same vertical line do not differ statistically (Tukey test, *P < *0.05). (**A**,**B**) Asterisks indicate significant differences (Tukey test, *P* < 0.05) among the *S*. *zeamais* SzSusc strain (i.e., the pyrethroid susceptibility pattern strain) and the weevils strains that were pyrethroid-resistant (i.e., the *S*. *zeamais* SzPyrSel strain) or pyrethroid-tolerant (i.e., the *S*. *oryzae* SoPyrTol and SoPyrR strains) when not challenged by the insecticide exposure (i.e., control treatment).

Multivariate analysis of variance for the walking parameters recorded for the four weevil strains (i.e., the maize weevil strains SzPyrSel and SzSusc and the rice weevil strains SoPyrR and SoPyrTol) revealed significant differences only for the strains (df_num/den_ = 24/782; Wilks’ lambda = 0.7848; *F* = 2.34; *P* < 0.0003) and the interactions between strains and insecticides (df_num/den_ = 12/592; Wilks’ lambda = 0.6637; *F* = 8.28; *P* < 0.001). Significant locomotory alterations induced by the insecticide treatments were found for walked distance (*df* = 3; *F* = 2.46; *P* = 0.025) and walking time (*df* = 3; *F* = 5.49; *P < *0.001; Fig. 4). Insects from SzPyrSel and SoPyrTol strains walked longer distances (Fig. 4A) and faster (Fig. 4B) when challenged by the pyrethroid exposure. However, insects from the SoPyrR strain walked slower and for shorter distances when challenged with either insecticide (e.g., deltamethrin or lambda-cyhalothrin) (Fig. 4A,B). The pyrethroid-susceptible standard strain of maize weevil (i.e., SzSusc) did not exhibit any significant modification in walking parameters with insecticide exposure (*P* > 0.05).

## Discussion

The frequent and indiscriminate use of synthetic insecticides for the control of stored product insect pests contributes to the selection of stored grain weevils (i.e., *Sitophilus* spp.) exhibiting high levels of pyrethroid resistance. Here, by completely sequencing and characterizing the *para*-orthologous sodium channel gene and by performing biochemical (e.g., synergism and detoxification enzymes) and behavioral (e.g., walking activities) assays with strains of rice and maize weevils, we recognized multiple and distinct mechanisms that conferred pyrethroid resistance in both species. The present investigation demonstrated for the first time the contribution of a *kdr* mutation (e.g., L1014F) to pyrethroid resistance among strains of the rice weevil. The L1014F mutation was not detected in Australian or Turkish rice weevil strains or in 14 Brazilian strains of maize weevil surveyed. By contrast, high pyrethroid resistance was detected in Brazilian strains of the maize weevil with the associated occurrence of another (super) *kdr* mutation (i.e., T929I), which apparently is a primary pyrethroid resistance mechanism among these strains.

Target-site insensitivity, increased (enzymatic) detoxification and behavioral changes are prominent mechanisms in insect resistance to pyrethroids. In terms of target-site insensitivity, drastic reductions in the sodium channel sensitivity to pyrethroids are often related to one or a few *kd*r mutations in the sodium channel gene^38–41^. These mutations and the associated pyrethroid resistance profoundly affect the management not only of human disease vectors (e.g., mosquitoes) but also of several important insect pests of agriculture and stored products, including grain weevils. The presence of the *kdr* mutation L1014F in all investigated Latin American strains of rice weevil (i.e., Brazil, Argentina and Uruguay) and the absence in strains from Turkey and Australia might be due to high selection pressure exerted by DDT (up to the 1980’s) and pyrethroid insecticide applications since the 1980’s in Latin America^42,43^. However, the absence of the L1014F mutation among Brazilian maize weevils is intriguing. Although both weevil species have overlapping distributions in southern part of Brazil and are subjected to similar selection pressures^29^, their evolutive and demographic histories are independents as the two weevil species split around 8.5 million years ago^30,31^, suggesting that the mutations based resistance in the *Sitophilus* species was generated by independent evolution events.

Lending more complexity to the management of maize weevils in the Brazilian scenario, the full-length molecular characterization of the laboratory-selected pyrethroid-resistant and the two other originally field-collected strains with pyrethroid resistance revealed only the occurrence of the T929I mutation, which is considered a more potent form of resistance (i.e., *super-kdr*) by affecting interactions of both DDT and pyrethroids with the insect sodium channels^12,40,44^. Although the T929I mutation in pyrethroid-resistant insects has generally been reported in association with other *kdr* mutations^45–49^, the mutation is also reported alone in several pyrethroid-resistant insect species^27,50–53^. The T929I has previously been detected in low frequency in Brazilian field-collected strains of maize weevil^27^, but our results with the laboratory-selected pyrethroid resistant strain (i.e., SzPyrSel) indicated that the T929I mutation can be selected independently of other more common mutations (e.g., L1014F), causing an increased level (i.e., higher than 2500-fold) of pyrethroid resistance without causing any functional impairment to the sodium channel, as was initially proposed^45^.

Thus, our findings for the laboratory-selected pyrethroid-resistant maize weevil strain SzPyrSel confirm the high resistance levels conferred by the T929I mutation and support the results of previous electrophysiological studies demonstrating that this substitution in the sodium channel confers high insensitivity to DDT and several type I and type II pyrethroids^8,54,55^. Therefore, as the T929I mutation increases in frequency in Brazilian maize weevil strains, the management of this pest will be seriously compromised in the country. However, the decrease in pyrethroid resistance levels recorded for the Brazilian maize weevil strains (i.e., SzPyrR1 and SzPyrR2) after a few years of laboratory rearing without insecticide exposure raises questions about the costs and fixation of such a mutation under natural selection conditions.

In the present investigation, the rice weevil strains also exhibited metabolic-based resistance confirmed by the synergism bioassays and due to increased detoxification by cytochrome P450 and GST. Increased activity of detoxification enzymes, in the presence or absence of target-site alterations, is a well-known biochemical mechanism of insecticide resistance^56^ and the three enzyme groups investigated in our study are the most commonly involved in resistance to several insecticides in different insect species. In fact, increases in GST levels can attenuate pesticide-mediated oxidative stress^57^ and play an important role in insecticide resistance^58^. The involvement of monooxygenases in pyrethroid resistance has been clearly demonstrated^59^. The role of detoxification enzymes in pyrethroid resistance has not been previously investigated in the rice weevil; however, increased detoxification as a resistance mechanism has been explored among Brazilian strains of the maize weevil^20,25,33,60^ and increases in glutathione-S-transferase activity are associated with pyrethroid resistance. However, this resistance mechanism is apparently of secondary importance to altered target-site sensitivity^58^, which is consistent with our results for the maize weevil strain SzPyrSel, which did not exhibit increased detoxification activity.

Behavioral resistance to pyrethroids may also occur in addition to target site and metabolic resistance mechanisms. As an example, changes in locomotory activity are reported in the maize weevil^61–64^. Here, we detected that pyrethroid exposure led to an increase in walking activity in the two weevil strains (i.e., the *S*. *zeamais* SzPyrSel and the *S*. *oryzae* SoPyrTol). Similar increases in walking activities were previously described for the maize weevil strains SzPyrR1 and SzPyrR2 when exposed to the pyrethroid cypermethrin^65^. By increasing walking activity, insects may quickly evade contaminated areas, which is a common strategy to minimize exposure to natural and synthetic insecticides^33,34,61,64,66,67^. Indeed, reductions in walking activities were recorded for individuals of the pyrethroid-resistant rice weevil strain (SoPyrR) when exposed to pyrethroids, suggesting distinct contributions of such behavioral mechanisms to pyrethroid resistance among stored grain weevil species.

Our results confirmed the resistance to pyrethroids in Brazilian strains of the maize weevil, as previously reported^20,68^ and identified pyrethroid resistance among strains of the rice weevil. Indoxacarb resistance is also reportedly associated with pyrethroid resistance in maize weevils, suggesting potential metabolic-based cross-resistance between these compounds^33^ because the mutations found here are not part of the indoxacarb binding site on sodium channels^69–71^. Furthermore, the moderate levels of resistance to neonicotinoids detected in the maize weevil strains were not expected because these two compounds are not used as grain protectants in Brazil, although they are reported in stored product pest insects^72^. Although in the initial stages, the resistance to other groups of insecticides adds to the justified concern with the phenomenon among stored grain pest species (and in grain weevils in particular), further highlighting the requirement for alternative management approaches in storage facilities.

In summary, our results provide evidence of multiple mechanisms of pyrethroid resistance among Brazilian strains of maize and rice weevils with the prevalence of altered target-site sensitivity. Emerging resistance to different groups of insecticides, even to those compounds not used in stored products and therefore likely from field use, is an additional challenge for the sustainable management of these pest species in the future. Nonetheless, more effort is required to recognize the broad patterns of co-occurrence of resistance mechanisms among grain weevils and other economically important stored products pests.

## Materials and Methods

### Insect populations

Strains of the maize weevil *S*. *zeamais* were collected from representative maize producing regions in Brazil (Table S1). Additionally, we also used three laboratory strains with levels of pyrethroid resistance previously described elsewhere^20,25,60,73,74^. We used the laboratory strain originally collected from the Maize and Sorghum Research Center of the Brazilian Agriculture Research Corporation (EMBRAPA Milho & Sorgo, Sete Lagoas, state of Minas Gerais, Brazil), which is a standard pyrethroid-susceptible strain (named hereafter SzSusc), and two DDT and pyrethroid-resistant populations from the regions of Jacarezinho-PR (SzPyrR1) and Juiz de Fora-MG (SzPyrR2). All strains were reared in glass containers under controlled conditions (25 ± 2 °C, 70 ± 10% relative humidity, 14:10 h lighting regime [D:L]) on insecticide-free maize grains. Furthermore, we also used a laboratory strain that was selected for pyrethroid resistance (SzPyrSel), which was obtained from at least 50 live, unsexed individuals from each of the above mentioned strains (i.e., for a total of 1000 live insects) that were submitted to insecticide selection pressure for 15 generations using the pyrethroid insecticide deltamethrin. After this selection pressure, the resistance of the SzPyrSel strain to pyrethroids increased more than 2500-fold compared with that of the susceptible standard strain (i.e., SzSusc).

For the rice weevils, we used one strain that was originally collected in a rice storage facility from the county of Cascavel (state of Paraná, Brazil) and another one collected at the harbor of Santos (county of Santos, state of São Paulo, Brazil) in rice carriers containing grains originally produced in Argentinian rice fields. Because the individuals of the Cascavel strain were more susceptible to pyrethroids than those of the Argentinian strain, they are hereafter termed SoPyrTol (Cascavel) and SoPyrR (Argentina) strains. Additionally, for the molecular characterization, we also used rice weevil strains field-collected from 12 locations around the globe (i.e., Brazil [5], Australia [4], Turkey [2] and Uruguay [1]). Once collected, these materials were kept in 95% ethanol at −20 °C until used.

All the insect populations used were subjected to identification using molecular identification with species-specific primers designed in the cytochrome oxidase subunit I as described in^29^.

### Concentration-mortality bioassays

Concentration-mortality bioassays were performed with the maize and rice weevil strains using six insecticide formulations as follow: the pyrethroids deltamethrin (Decis, 25 g/L, Bayer CropScience, Belford Roxo-RJ, Brazil) and lambda-cyhalothrin (Karate Zeon, 50 g/L, Syngenta Proteção de Cultivos Ltda, Paulínia–SP, Brazil); the neonicotinoids imidacloprid (Evidence, 700 g/kg, Bayer CropScience, Belford Roxo-RJ, Brazil) and thiamethoxam (Actara, 250 g/kg, Syngenta Proteção de Cultivos Ltda, Paulínia–SP, Brazil); the spynosin spinosad (Tracer, 480 g/L, Dow Agro Sciences Industrial Ltda, Guaíra–SP, Brazil); and the oxadiazine indoxacarb (Rumo, 300 g a.i./L, DuPont do Brasil S.A., Barueri-SP, Brazil). The bioassays were conducted in a completely randomized design following previously described methods^33,75^. Briefly, the insecticide solutions (with distilled and deionized water as the solvent) were sprayed at a rate of 1 mL (insecticide) of emulsion on 200 g of maize grains placed in a rotary stainless steel container to homogenize the grains during the application. An artist’s airbrush (Sagyma SW440A; Yamar Brazil, São Paulo, SP, Brazil) coupled with an air pump (Prismatec 131A Tipo 2VC; Itu, SP, Brazil) was used for spraying the insecticide solutions at a pressure of 0.7 kgf/cm^2^. The grains were allowed to dry in the container for one h. Control grains were treated only with distilled and deionized water. Five replicates were used, each consisting of 20 g of insecticide-treated maize grains (placed in 20-mL glass vials) and 20 non-sexed adult weevils (<one-week-old). After 24 h of exposure, the mortality was recorded. Insects were considered dead when unable to walk when prodded with a fine hairbrush. At least six different insecticide concentrations were used to estimate each concentration-mortality curve.

### Synergism bioassays

The synergism bioassays were performed with the maize (i.e., SzPyrSel) and rice weevil (i.e., SoPyrR and SoPyrTol) strains that exhibited the highest pyrethroid resistance level in the concentration-mortality bioassays. In these synergism bioassays, we used the pyrethroid insecticides deltamethrin and lambda-cyhalothrin. The two insecticides were synergized following methods described by Ribeiro, *et al*.^20^ using three different compounds: triphenyl phosphate (an esterase inhibitor), diethyl maleate (a glutathione S-transferase inhibitor) and piperonyl butoxide (an inhibitor of cytochrome P450-dependent monooxygenases and esterases). Acetone was used to dissolve the synergists, and 1 mL of the synergist solutions (1 mg/mL) was applied to the inner walls of 20-mL vials and left to dry by rotation. Twenty unsexed adults were transferred to each vial and left in contact with the synergist for one hour before exposing the insects to insecticide-treated maize grains as described before. Because the mortality results obtained for individuals from the SzPyrSel strain did not allow any concentration-response curve (because of the high resistance level), we exposed the individuals of this strain to a pyrethroid concentration of 68 mg/kg for deltamethrin and 71 mg/kg for lambda-cyhalothrin, corresponding to 100-fold increases in the LD_50_ obtained for the pyrethroid susceptible standard strain (i.e., SzSusc).

### Cloning of sequences encoding the *para*-orthologous sodium channel gene

Total RNA was extracted from pools of 20–35 adult individuals (either maize or rice weevils) using Trizol (Thermo Fisher Scientific Inc., Waltham, MA, USA) and following the manufacturer’s instructions. Extracted RNA was further purified using DNA-free DNase treatment and removal reagent (Ambion) to remove genomic DNA. The quality and quantity of RNA pools were assessed by running an aliquot on a 1.2% agarose gel and also by spectrophotometry (NanodropTechnologies). The RNA, 5 μg, was then used for first strand cDNA synthesis using Superscript III and oligo(dT) (Invitrogen) according to the manufacturer’s instructions. The prepared cDNA was subsequently used in polymerase chain reaction (PCR) initially using degenerate primers designed against conserved regions of sequences of the *para* sodium channel gene from different insect species (*Musca domestica* AY834743; *Drosophila melanogaster* M32078.1; *Brassicogethes aeneus* KJ699123.1; *Tribolium castaneum* XM962937 and XM962927.2; *Leptinotarsa decemlineata* AF114489.1; *Dendroctonus ponderosae* XM_019909635.1 and *Blattella germanica* BGU73584). Subsequent PCR was performed using specific primers designed based on the fragments sequenced (Fig. 1A and Table 4). Amplification of cDNA fragments by PCR was performed in a 25 μL mixture including 1 μL of template DNA, 1 μL of each primer (10 μM), 12.5 μL of GreenTaq (Fermentas) and 9.5 μL of sterile distilled water. PCR cycling consisted of an initial denaturation at 94 °C for 90 s, followed by 35 cycles of 94 °C for 30 s, 48–58 °C for 45 s and 72 °C for 90–120 s and a final cycle of 7–10 min at 72 °C.

**Table 4:** Oligonucleotide primers used in this study.

Fragment	Name	Sequence	Length (pb)
a	DgNF4 DegenR5 *SVGCF1* *SVGCR1*	AGHTTGTTCCGWCCBTTYAC GCTCGYACTACCCTRAAYGTTCT *AGTTTGTTCCGTCCCTTCAC* *AGCCTCAACACCCTAAATGTTCT*	620
b	DegenF7 DgNR9 *SVGCF2*	AATCAGCIGTAAAGTIATGGC HGCYTCHCGHADNGCYTCYT *AATCAGCAGTGAAGGTGATGGC*	1610
c	DegenF9 *SZ4* ^(^ * ^)^ *SVGCF3* *SZ3* ^(^ * ^)^	GGCCATTGTYGCCATGTCDTA *CTTGAAGATCCAAAGTTGCTC* *GCCATTGTCGCCATGTCGTACGA* *GAGCGGACAAACTTGAAGATC*	1610
d	*SZ1* ^(^ * ^)^ *SZ2* ^(^ * ^)^ *SVGCR2*	*ACCCTGAACTTATTGATATCC* *TGAACTTATTGATATCC* *AACCAAATATAATAAAGAATACGAAG*	2000
e	DegenF2 DegenR3 *SVGCF4* *SVGCR3*	CCITTYTGGCARGGITGG TACATRTCRTARTCRTCRTC *CCTTTCTGGCAAGGCTGG* *TACATATCGTAATCGTCGTC*	2020
f	DegenF4 *SZend R1* *SVGCF5*	GGITGGATHCARATHATGAA *GACATCCGCTGATCGTGAG* *GGATGGATTCAAATTATGAA*	1650

*From Araujo, *et al*.^27^; Specific primers are in *italic*.

PCR products were separated by agarose gel (1.2%) electrophoresis in 1× TBE buffer. The desired DNA fragments were recovered from gel slices using the Wizard SV gel and PCR clean up System from Promega. The isolated fragments were cloned using StellarTM Competent Cells (Clontech, SP, Brazil) according to the manufacturer’s instructions. Plasmid DNA was sent to Macrogen Inc. (Macrogen Inc., Seoul, S. Korea) for sequencing using standard T3/T7 primers. To amplify the region IIS4-IIS6 of the fragment encompassing the L1014F and T929I mutations, individual genomic DNA was used in a two-step nested PCR with the specific primers SZ1 and SZ3 used in primary PCR and SZ2 and SZ4 used in secondary PCR and 15–20 individuals from each population were used to determine the frequency of each mutation.

### Biochemical assays

Biochemical assays for general esterases, glutathione-S-transferase (GST) and cytochrome P450 were conducted following Hemingway, *et al*.^76^ with some modifications. Five replicates of 20 adult insects each were used for these assays. The insects were homogenized in 3.0 mL of phosphate buffer (0.1 M, pH 7.5) and Triton-X100 (0.3%). The homogenates were centrifuged at 10,000 rpm and 4 °C for 15 min and the resulting supernatant was used as the enzyme source. General esterase activity was determined in 96-well microplates using α-naphthyl acetate as substrate^77^. Three aliquots of 50 µL of enzyme preparations were pipetted into separate wells. The reaction started with the addition of α-naphthyl acetate 0.3 mM solution. After 15 min of incubation at 30 °C, 50 µL of dizablue laurylsulfate of sodium (DBLS) solution was added to each well to stop the reaction. The mixture was left for 15 min at room temperature, and the enzyme activity was read at 600 nm. Absorbance levels were compared with a standard curve of absorbance for known concentrations of α-naphthol. GST activity was measured using 1-chloro-2,4-dinitrobenzene (CDNB) (Sigma Aldrich) as the substrate^78^ by adding into a 2.5 mL quartz cuvette 1760 µL of phosphate buffer (0.1 M, pH 7.5), 200 µL of enzyme solution, 20 µL of 150 mM CDNB solution, and 20 µL of 150 mM GSH solution. Absorbance was measured continuously every 30 s during 90 s at 340 nm using a spectrophotometer (UV 1800; Shimadzu Corp., Kyoto, Japan).

The cytochrome P450 activity was measured using the haem-peroxidase assay, which is an indirect measure of cytochrome P450^79^. The haem content of the weevil samples was measured by transferring three aliquots of 20 µL of enzyme preparations into separate wells and adding 60 µL of potassium phosphate buffer (1M, pH 7.2), 200 µL of TMBZ solution, and 25 µL of hydrogen peroxide (3%). The mixture was left for 30 min at room temperature. Absorbance was read at 650 nm, and the content values were determined via standard curve of absorbance for known concentrations of cytochrome C.

### Behavioral bioassays

Behavioral bioassays were conducted in arenas fully treated with insecticide (deltamethrin and lambda-cyhalothrin), as previously described elsewhere for other insecticides^33,68,74,80^. Control treatments consisted of acetone only. Briefly, filter papers were impregnated with 1 mL of insecticide solution at a concentration corresponding to the determined LD_50_ of the susceptible standard strain (i.e., SzSusc or SoPyrTol) and after drying for 20 min were placed in Petri dishes (90 mm in diameter). The inner walls of each Petri dish were coated with Teflon PTFE (DuPont, Wilmington, DE) to prevent insect escape. The movement of each insect within the arena was recorded for 10 min using an automated video tracking system equipped with a charge-coupled device (CCD) camera (ViewPoint Life Sciences Inc., Montreal, CA). The parameters recorded were walked distance (cm), velocity (cm/s), and time spent walking (s) in the arena. Twenty insects were used for each population and insecticidal treatment. In each trial or replicate, the filter paper was replaced. The side of the arena on which the insect was released was randomly chosen in each trial.

### Statistical analyses

Concentration-mortality data were subjected to probit analysis^81^, and 95% confidence intervals for resistance ratios were estimated following Robertson, *et al*.^82^ and considered significant when not including the value 1. Differences between synergized and unsynergized insecticides for a given population were analyzed using one way ANOVA followed by Tukey’s post hoc test. The overall results for walking activities were subjected to a two-way (insecticide treatment x population) multivariate analysis of variance (PROC GLM with MANOVA statement)^81^. The individual walking traits were subsequently subjected to a two-way (univariate) analysis of variance and Tukey’s honestly significant difference (HSD) test (*P* < 0.05) when appropriate (PROC GLM)^81^. The assumptions of normality and homoscedasticity were checked (PROC UNIVARIATE)^81^ and no data transformation was necessary.

### Ethical approval

All applicable international, national, and institutional guidelines for the care and use of animals were considered in the present investigation.

### Informed consent

All the authors of this manuscript accepted that the paper is submitted for publication in the *Scientific Reports* journal, and report that this paper has not been published or accepted for publication in another journal, and it is not under consideration at another journal.

## Electronic supplementary material

Supplementary Information

## Data Availability

All data generated or analysed during this study are included in this published article (and its Supplementary Information files).
