# Supplementary material for: Diversity and convergence of mechanisms involved in pyrethroid resistance in the stored grain weevils, Sitophilus spp
Source: Sci Rep. 2018 Nov 5;8:16361. doi: 10.1038/s41598-018-34513-5 (PMC6218525; doi:10.1038/s41598-018-34513-5)
Supplement: Supplementary file 1 — Supplementary Information [file 41598_2018_34513_MOESM1_ESM.pdf]

Diversity and convergence of mechanisms involved in pyrethroid resistance in the stored grain weevils, *Sitophilus* spp.

K. Haddi[a,b]*, W. R. Valbon[a], L. O. Viteri Jumbo [a], L. O. de Oliveira[c], R. N. C. Guedes[a,d], E. E. Oliveira[a]*

[a]*Departamento de Entomologia, Universidade Federal de Viçosa, Viçosa, MG 36570-900, Brasil*

[b]*Science without Border Program, Associate Researcher, Programa de Pós-Graduação em Entomologia, Universidade Federal de Viçosa, Viçosa, MG 36570-000, Brasil*

[c]*Departamento de Bioquímica e Biologia Molecular, Universidade Federal de Viçosa, Viçosa, MG 36570-900, Brazil*

[d] *USDA Agricultural Research Service, San Joaquin Valley Agricultural Sciences Center, Parlier, CA 93648, USA*

Supplementary material

Table S1. Strains of maize and rice weevils (*Sitophilus* spp.) with their country of origin, state, and conditions under this study.

**Strains**	**Species**	**Country**	**State**	**Conditions**
Amambai	*S. zeamais*	Brazil	Mato Grosso do Sul	Rearing
Balsas	*S. zeamais*	Brazil	Maranhão	Rearing
Barreiras	*S. zeamais*	Brazil	Bahia	Rearing
Canarana	*S. zeamais*	Brazil	Mato Grosso	Rearing
Cristalina	*S. zeamais*	Brazil	Goiais	Rearing
E.S. Pinhal	*S. zeamais*	Brazil	Sao Paulo	Rearing
Ipojuca	*S. zeamais*	Brazil	Pernambuco	Rearing
Iragassu	*S. zeamais*	Brazil	Pernambuco	Rearing
Jacarezinho (SzPyrR1)	*S. zeamais*	Brazil	Paranà	Rearing
Juiz de Fora (SzPyrR2)	*S. zeamais*	Brazil	Minas Gerais	Rearing
Piracicaba	*S. zeamais*	Brazil	Sao Paulo	Rearing
Sao João	*S. zeamais*	Brazil	Paranà	Rearing
Teresina	*S. zeamais*	Brazil	Piaui	Rearing
Xapuri	*S. zeamais*	Brazil	Acre	Rearing
Sete Lagoas (SzSusc)	*S. zeamais*	Brazil	Minas Gerais	Rearing
Cascavel (SoPyrTol)	*S. oryzae*	Brazil	Paranà	Rearing
Viçosa	*S. oryzae*	Brazil	Minas Gerais	Rearing
Viçosa1	*S. oryzae*	Brazil	Minas Gerais	95% ethanol
Volta Redonda	*S. oryzae*	Brazil	Rio de Janeiro	Rearing
São Borja	*S. oryzae*	Brazil	Rio Grande do Sul	Rearing
Porto Nacional	*S. oryzae*	Brazil	Tocantins	Rearing
SoPyrR	*S. oryzae*	Argentina	-	Rearing
Uruguay	*S. oryzae*	Uruguay	-	Rearing
QQSO1537	*S. oryzae*	Australia	Moura Queensland	95% ethanol

QQSO1543	*S. oryzae*	Australia	Springsure Queensland	95% ethanol
QTSO1	*S. oryzae*	Australia	Tasmania	95% ethanol
QQSO16	*S. oryzae*	Australia	(South Australia	95% ethanol
Trukey1	*S. oryzae*	Turkey	-	95% ethanol
Turkey2	*S. oryzae*	Turkey	-	95% ethanol

All the strains reared in the laboratory were tested for insecticides susceptibilities

Figure S1. Alignment of the deduced amino acid sequences of *S. zeamais* and *S. oryzae* with *Musca domestica para* sodium channel proteins. Dots represent identical residues, and dashes indicate gaps introduced to obtain optimal alignment. Numbers on the right indicate the amino acid positions in each protein. The six transmenbrane segments (S1–S6) in each of four homologous domains (I–IV) are highlighted in grey. The positions of the encountered mutations (T929 and F1014) are boxed.

```
S.zeamais   ------------ASLFRPFTRESLAAIEARIAEEHAKQKELEKKRAEGEPGFGRRKKKKEIRYEDEDEDEGPQPDQTLEQGLPLPVRLQGNFPPELASTPLEDIDPFYSNQMTFVVISKGKDIFRFSATNALWILDPFNPIRRVAIYILVHPLFSLFIIT
S.oryzae    ----------------------------....................................................................................................................................
M.domestica MTEDSDSISEEER..........LLQ..Q....-.E......R...A---------EGEQ...D...........P.....V.I...M..S...................VL................SK.M.L..........................

S.zeamais   TILVNCILMIMPTTPTVESTEVIFTGIYTFESAVKVMARGFILQPFTYLRDAWNWLDFVVIALAYVTMGIDLGNLAALRTFRVLRALKTVAIVPGLKTIVGAVIESVKNLRDVIILTMFSLSVFALMGLQIYMGVLTQKCIKVFPMDGSWGNHTDENWER
S.oryzae    ...V.............................................................................................A......................................................L.......
M.domestica ...T.......................................C..............................................................................................R..L......L.....FL

S.zeamais   FNQNETNWYFDEEKGEIPLCGNSSGAGQCKPGYMCLQGYGDNPNYGYTSFDTFGWAFLSAFRLMTQDYWENLYQLVLRSAGPWHMLFFIVIIFLGSFYLVNLILAIVAMSYDELQKKAEEEEAAEEEAIREAEKAAQAKQDRADARAAAAEEAARVAAA-
S.oryzae    ..I....................................................................................................N...................I...................................-
M.domestica H.S.SS..FTENDGESY.V...V......GED.V....F.P....D.....S...............F..D...H..QA......................................................E..A..AAKLEE..NV.AQ..QD..DA

S.zeamais   -GAGSTDIVKSPSDFSCQSYELFVNQPKGTDDNNKEKMSIRSEGLDSVSE--QRR----IPTNPTKMRKVSAASLSLPGSPFNLRRGSRGSHQFTARNNRRMV-APPGDRKPLVLSTYLDAQEHLPYADDSNAVTPMSEENGAMVVPMYYANLGSRHSSY
S.oryzae    -..............................................-------------------------...............................-........................................................
M.domestica AA.ALHPEMAKSPTY.........GGE..N............VEVE.ESVSVIQRQPAPTTAPA..V....TT................S..KY.I..G.GRFGI.GS.......Q..Q...Q....................II..A..C.........

S.zeamais   TSHASRMSYTSHGDLLGGFGGNGKV-MTKETQLLMRSMRN---------GPAMTGNNFTEFTHKPKMGD-YDGPTSQLCEKMKALDNPFIDNISQRQTVVDMKDVMVLNDIIEQAAGREITDGEHGVAVYYFSANNEEEEEEEEPTMKERILENTLKIID
S.oryzae    .........................-..............---------....................-..........................................................................................
M.domestica ...Q..I...........MAAM.AST....SK.RS.NT..QSIGAATNG.SSTA.GGYPDAN..EQRDYEMGQDYTDEAG.I.HH.....EPV-.T.......................SRA..R.-----------.DDD.DG..F.DIA..YI..GEI

S.zeamais   MFCVWDCCWCWLQIQKYVALVVFDPFVELFITLCIVVNTLFMALDHHNMDPDLEKALKSGNYFFTATFMIEATMKLIAMSPKFYFQEGWNIFDFIIVALSLLELGLEGVQGLSVLRSFRLLRVFKLAKSWPTLNLLISIMGRTMGALGNITFVLCIIIFI
S.oryzae    .....................................................................M..........................................................................................
M.domestica .........V..KF.EW.SFI..................M...M...D.N.E...V............A...S...M.....Y.............................................................................

S.zeamais   FAVMGMQLFGKNYWDNVDRFPDHQMPRWNFTDFMHSFMIVFRVLCGEWIESMWDCMHVGDVSCIPFFLATVVIGNIVVLNLFLALLLSNFGSSSLSAPTADNDTNKIAEAFERIARFINWIKANCMHFAKLVRFKLTNQISDQPPDTRDGGLDIQDDEIL
S.oryzae    ................................................................................................................................................................
M.domestica ............I..HK...K..EL...............................Y......................................................N.....K..V.R.IADCF..I.N..........SEHG.NE.ELGH...M

S.zeamais   ADGIIFKDKKSPKDKFEVTIGDGMEFTIHGDSKTNLKRGKNMNNINNKNKTIGNSILDHGDF---------IGHGDDDEISNKSYGSHKNR-FKEESHKGSADILDDQEEKRDASKEELGIDEEIEEDECDCQGPLDEDLIIDAATEDIILDEYSADCFP
S.oryzae    ..............................................................---------.....................................................M...................................
M.domestica G..L.K.GM.-GETQL..A............M.N.KPKKSKFI---.NTTM.....NHQDNRLEHELNHRGLSIQ...TA.IN........PFKD.......ETIE-G.....V...D..L...LD.EAEGDE.Q..G.I..H.QND.E.I.D.P.....

S.zeamais   EPCYKKFPFLAGDEDSPFWQGWGNLRYKTFQLIENKYFETAVITMILLSSLALALEDVHLSQRPILQDILYYMDRIFTVIFFFEMLIKWLALGFQKYFTNAWCWLDFVIVMVSLINFIASLCGAGGIQAFKTMRTLRALRPLRAMSRMQGMRVVVNALVQ
S.oryzae    ................................................................................................................................................................
M.domestica DSY.....I.................L....................M............PD..VM.................L...........KV...............L...LV.VWSGLNDIAV.RS............V..WE..K........

S.zeamais   AIPSIFNVLLVCLIFWLIFAIMGVQLFAGKYYKCVDNNKTTLSWEIIPDYNACKAENYTWDNSRMNFDHVGKAYLCLFQVATFKGWIQIMNDAIDSRELHKQPIRETNIYMYLYFVFFIIFGSFFTLNLFIGVIIDNFNEQKKKAGGSLEMFMTEDQKKY
S.oryzae    ................................................................................................................................................................
M.domestica ...............................F..K.G.D.V..H....NR....S.....E..A.......N..........................VD............................................................

S.zeamais  YNAMKKMGSKKPMKAIPRPRWRPQGIVFEIVTNKKFDMFIMLFIGLNMLTMTMDHYQQKETFTKVLDCLNMIFIVIFTSECLMKIFALRYHYFTEPWNLFDLVVVILSILGLVLSDIIEKYFVSPTLLRVVRVAKVGRVLRLVKGAKGIRTLLFALAMKTF
S.oryzae   ......................................................................................................L..........................................................
M.domestica............L...........A.......D....II..........F..L.R.DAS.AYNN...K..G..V...SG...L.........K....................................................................

S.zeamais   SLPALFNICLLLFLVMFIFAIFGMSFFMHVKDKSGLDDVYNFGQSMILLFQMSTSAGWDGVLDGIINEEDCKQPNNEIGETGNCGNSTIGIAFLLSYLVISFLIVINMYIAVILENYSQATEDVQEGLTDDDYDMYYEIWQQFDPDGTQYIRYDQLSDFL
S.oryzae    ................................................................................................................................................................
M.domestica ...............................E...INA.........................A........DP.D.DK.YP....SA.V..T....................................................E...........E..

S.zeamais   DVLEPPLQIHKPNKYKIVSMDIPICKGDLMYCVDILDALTKDFFARKGNAIDETAELSEVQPKPNEVGYEPVSSTLWRQREEYCARLIQNAWRKHKRLRGATDQ-----------SGDEGDMGDGDGDGDGNDE-------------------ID-----
S.oryzae    ........V...............................................................................................-----------SGDE...............-------------------..-----
M.domestica .................I...M...R..M....................P.E..G.IG.IAAR.DTE..D...............K.......RY.NGPPQEGDEGEAAGGEDGAE.G..EG.S.G.G.G.D.GGSATGATAAAAGATSPSDPDAGEADG

S.zeamais    ---------GEADGELEARQTAV------LVERNGHKVVIHSRTPSISSRSADV 2043
S.oryzae     ---------..............------......................... 2008
M.domestica  ASVGGPLSP.CVS.GSNG.....LVESDGF.TK..........S...TSRT... 2108
```
